# Monitoring the Snowpack Volume in a Sinkhole on Mount Lebanon using Time Lapse Photogrammetry

**DOI:** 10.3390/s19183890

**Published:** 2019-09-09

**Authors:** Charbel Abou Chakra, Simon Gascoin, Janine Somma, Pascal Fanise, Laurent Drapeau

**Affiliations:** 1 Laboratoire de Télédétection, Centre de Recherche en Environnement-Espace Méditerranée Orientale, Université Saint-Joseph, Beirut BP 17-5208, Lebanon; 2 Arab Union of Surveyors, Beirut BP 9300, Lebanon; 3 Faculty of Engineering, Université Libano-Canadienne, Aintoura BP 32, Lebanon; 4 Centre d’Etudes Spatiales de la Biosphère (CESBIO), Université de Toulouse, CNES/CNRS/INRA/IRD/UPS, 31401 Toulouse, France

**Keywords:** snow, time-lapse camera, photogrammetry, structure-from-motion, water resource

## Abstract

Lebanon has experienced serious water scarcity issues recently, despite being one of the wealthiest countries in the Middle East for water resources. A large fraction of the water resources originates from the melting of the seasonal snow on Mount Lebanon. Therefore, continuous and systematic monitoring of the Lebanese snowpack is becoming crucial. The top of Mount Lebanon is punctuated by karstic hollows named sinkholes, which play a key role in the hydrological regime as natural snow reservoirs. However, monitoring these natural snow reservoirs remains challenging using traditional in situ and remote sensing techniques. Here, we present a new system in monitoring the evolution of the snowpack volume in a pilot sinkhole located in Mount Lebanon. The system uses three compact time-lapse cameras and photogrammetric software to reconstruct the elevation of the snow surface within the sinkhole. The approach is validated by standard topographic surveys. The results indicate that the snow height can be retrieved with an accuracy between 20 and 60 cm (residuals standard deviation) and a low bias of 50 cm after co-registration of the digital elevation models. This system can be used to derive the snowpack volume in the sinkhole on a daily basis at low cost.

## 1. Introduction

Lebanon is one of the wealthiest countries in the Middle East for water resources. Despite this, the people in Lebanon are frequently affected by water restrictions, especially during the dry summer season. In addition, the water demand is increasing, due to the growth of the native population and the large number of Middle Eastern refugees received in recent years [1].

In Lebanon, precipitation is enhanced by the Lebanon and Anti-Lebanon mountain ranges, which run parallel to the Mediterranean coast. An important fraction of the precipitation falls as snow on the Lebanese mountains, and the snow covers around 25% of the total Lebanese territory in winter [2,3,4,5]. Furthermore, it is estimated that approximately 30% of the national water resource originates from seasonal snow melt [4]. However, the seasonal evolution of the snowpack remains poorly known, as there is currently no operational snow national monitoring program [6,7]. Continuous and systematic snow observations are urgently required to improve water management planning [6,7].

The upper area of Mount Lebanon is a large upper cretaceous carbonate plateau [8]. A unique aspect of Mount Lebanon snow hydrology is related to its karstic features like sinkholes [9]. These basin-shaped hollows typically have a diameter of a few tens of meters or more, and a depth of a few meters. These sinkholes are closed and form natural collectors for rainfall and meltwater for groundwater recharge. In addition, due to their hollow shape, sinkholes on Mount Lebanon form natural traps for snow for several reasons [3,6]. First, sinkholes are preferential areas of wind-drifted snow deposition, due to the localized drop in wind speed on their lee side. Second, they isolate the snowpack from the surface atmospheric turbulence, which reduces the melting and sublimation rates. As a result, sinkholes on Mount Lebanon help delay the low flow period in downstream areas by providing meltwater late in summer [6]. This significant hydrological contribution has motivated the implementation of an observation system that monitors the evolution of the seasonal snowpack in a sinkhole.

From a water management perspective, the snow water equivalent (SWE) is the key variable to monitoring [10]. However, existing automatic SWE measurement devices are costly or difficult to install in rugged terrain. On the other hand, SWE variability is closely related to the variability of snow depth, therefore, snow depth monitoring is useful in hydrological studies [11,12]. In addition, bulk snow density can be simulated with reasonable uncertainty using empirical formulas or a snowpack model [13]. However, snow depth can be measured using sonic gauges in automatic weather stations. As sonic snow gauges provide only a point-scale measurement, given the limited footprint of the reflected echoes, this kind of device is not adapted to snow monitoring in sinkholes over Mount Lebanon where snow depth can vary from 0 to 15 m within a few tens of meters, due to their specific morphology. Spatially-distributed and accurate observations of snow depth can be obtained at low cost from drone photogrammetry [14]. However, the temporal frequency of drone surveys in Lebanon is often limited by the meteorological conditions or military restrictions. Another option is to use very high resolution stereo satellite images [15]. However, the acquisition of stereoscopic images has proven to be difficult due to the frequent cloud cover in winter and the fact that very high resolution satellites (in this case, Pléiades) are often tasked by the military, who have priority over the region. Finally, terrestrial lidar scanners are very efficient to determine the snow surface topography, but these devices are very costly and cannot be deployed in the field for continuous monitoring [16].

Terrestrial time-lapse photography has been increasingly used in environmental studies, including for snow cover monitoring in small- and medium-sized catchments [17,18,19]. This new technique is not expensive and can be easily implemented in mountain environments for snow studies [20,21]. However to the best of our knowledge, this method has only used to determine the extent of the snow cover and other derived metrics, like snow cover duration or the snow melt-out date. Revuelto et al. [21] used the snow cover area retrieved from time-lapse camera images to perform backward snow depth reconstruction. However, this approach relied on a degree-day model that was subject to uncertainties and required local weather data. On the other hand, photogrammetry is now widely used to generate three-dimensional (3D) models of the land surface, using multi-view images from digital cameras, in particular due to the development of structure-from-motion (SfM) algorithms [22,23,24,25,26]. For instance, Mertes et al. [27] used SfM from oblique terrestrial and aerial images to map glacier elevation changes.

Here, we present a new system using low cost digital cameras to monitor the snowpack volume in a sinkhole in Mount Lebanon. Three time lapse cameras were employed to derive a 3D model of the snow surface on a daily basis. We also evaluate the method using topographic surveys with a total station.

## 2. Study Area

**Figure 1:**
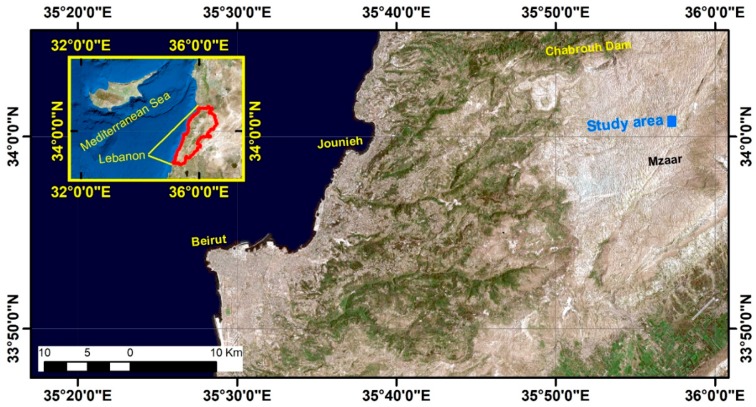
Location map.

The study area is located on the high plateau of Mount Lebanon (Figure 1). This karstic Cenomanian geological formation is punctuated with hundreds of sinkholes at elevation, ranging between 1700 and 3000 m. In this area, the seasonal snow can persist over six months [4]. For this study we focus on a pilot site at 2300 m in the Jabal el Dib region (Ouyoun el Simane-Kfardebian). This site is a sinkhole of about 16 m depth, 100 m wide, and 170 m long, located near the ski resort of Mzaar Kfardebian (35°51′28″ E; 33′58’53″ N). The bottom of the hole has an oval shape, with a diameter ranging from 30 to 36 m. There is a very sparse vegetation cover in the studied sinkhole (herbaceous and small shrubs), but most of the surface is covered with rocks and bare soil, including the floor and the walls of the sinkhole. Since 2010, the site has been equipped with an automatic weather station [7]. There is also a geodetic point very close to the sinkhole, which, together with the buildings of the ski center, facilitates geodetic surveys.

## 3. Method

### 3.1. System Setup

**Figure 2:**
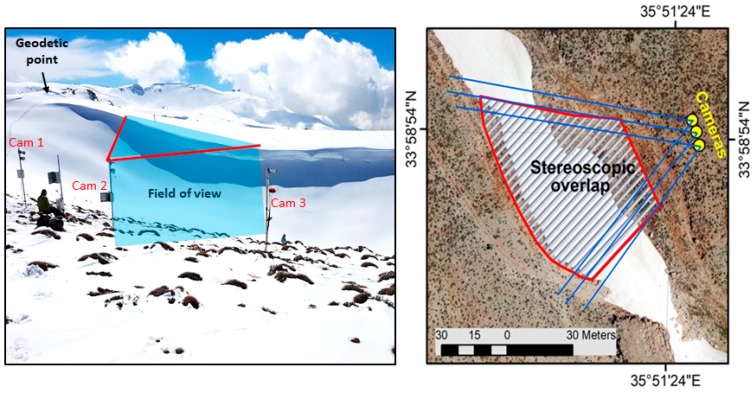
Setup: (**a**) Terrestrial view of the setup; (**b**) Drone ortho-image of the study area, taken on 24 April 2018 with 100 m as flight altitude.

For this study, three cameras were installed to image a surface area of about 4500 m^2^, with stereoscopic overlap around 80 % (Figure 2). The cameras were installed facing the dominant wind direction, in order to face the snow cornice, which is formed every year by wind drift (Figure 2). Each camera was mounted on 2 m height galvanized pole, fixed in a concrete block, which was poured within the soil (Figure 2). The system was designed to resist harsh conditions, including strong gusts in winter and to require minimal maintenance.

The spacing distance between each camera was determined empirically by taking a dozen photographs of the site, from different location, and testing different combinations. We found that an approximate spacing of 5 m between each camera allowed a good resolution of the surface topography. The cameras were set up with a downward inclination of the focal plane of about 30° from the zenith. We used three Canon PowerShot A1400 cameras (Konka Group Co., Ltd., Shenzhen, China), with a resolution of 4608 by 3456 pixels and a focal length of 5 mm. The cameras were connected to a Campbell CR200 data-logger and powered by a 12 V lithium battery. The CR200 was only used to switch on and off the cameras at fixed times. A DC-to-DC converter was installed within each camera case to convert the 12 V voltage from the CR200 to 3.6 V. The Canon Hack Development Kit (https://chdk.fandom.com/) was used to trigger photograph acquisition in automatic mode when the camera was switched on. The photographs were saved in the 32 Gb SD cards of each camera. The system was programmed to take three triplets of photographs per day at 08:00, 10:00, and 12:00 local time (UTC + 2 h). Morning acquisitions were preferred given the westward orientation of the cameras. The acquisition period started on 15 March 2015, and the system worked smoothly until complete snow ablation on 11 July 2015. However for this study, we only processed the images that were acquired on six different days during this period, when field validation surveys were conducted (see below).

### 3.2. Photogrammetric Processing

The snow volume in the sinkhole was computed by differencing a digital elevation models (DEM) of the snow-covered scene (snow-on DEM) with a DEM of same area without snow (snow-off DEM). To complete this operation, it was important that both DEMs are well aligned (co-registered). Hence, we defined two experiments, which differ by the method in co-registering the snow-on with the snow-off DEM. In addition, we also evaluated the impact of the source imagery to compute the snow-off DEM. The three experiments (Exp. 1, Exp. 2 and Exp. 3) are detailed below and summarized in Table 1.

To compute the DEMs, we used Agisoft Photoscan Professional to generate a time-stamped DEM, from three simultaneous photographs, taken by the time-lapse cameras. We selected Agisoft Photoscan because it is a state-of-the-art photogrammetric. The software can also run in batch mode, which integrates the SfM workflow with a user-friendly interface, but also because it can be used in batch mode through a Python application programming interface (API) to automate future processing. Numerous studies have successfully utilized Agisoft Photoscan in this manner [22,23,24,25,26,28,29,30]. We used the images that were acquired at 08:00 local time. Agisoft was configured to produce a DEM at 10 cm horizontal resolution. The coordinates of the cameras were surveyed with a dGPS and provided as input data to Agisoft. For each date, this process also allowed the generation of an ortho-rectified image of the scene from the photographs of the three cameras using the maximum of intensity at each pixel among the three images. This ortho-image was used in the co-registration step below.

**Figure 3:**
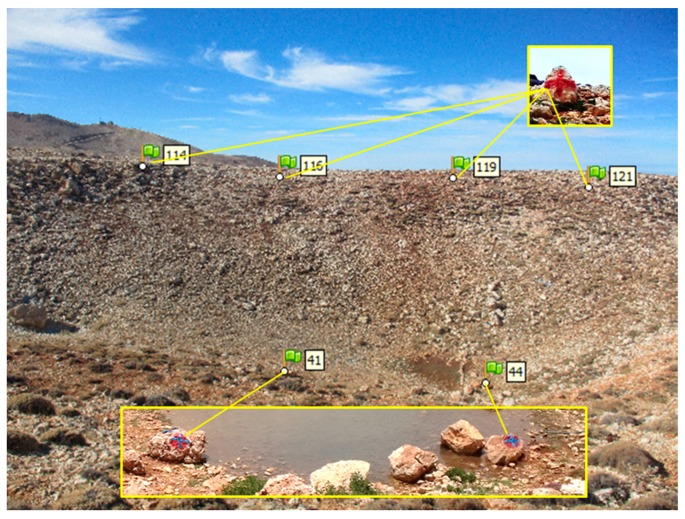
Ground control points (GCPs) distribution in the scene. These GCPs were used to reference the snow-off DEM from the camera photographs and to reference the snow-on digital elevation models (DEMs) in Exp. 1.

We used six ground control points (GCPs) in the scene to generate the snow-off DEM with Agisoft for all the dates as it is generally done to allow a good registration of the DEM to the spatial reference system of the study area (Exp. 1). The GCPs (Figure 3) were measured by total station (Pentax R-1505N) in the Lebanese stereographic projection system. The criteria for the establishment of the GCPs were that the GCPs should be well-distributed across the scene and located on a spot that is snow-free on most photographs (Figure 3). Yet, the GCPs could not be used for the first validation date (8 May 2015) as they were covered by snow. Only four GCPs out of six could be used on 20 May 2015 for the same reason.

However, GCPs must be manually marked by a visual inspection of the images, while, for this study, our aim was to develop an automatic method to process time series of daily image chunks. Hence for Exp. 2 and 3, we generated the snow-on DEMs, without GCPs. We used an automatic method to align each snow-on DEM with the snow-off DEM to compensate for the lack of GCPs. We used the iterative closest points (ICP) algorithm to register all the snow-on DEMs onto a snow-off DEM [31]. This algorithm finds a transformation matrix (translation, rotation, scale) that constrains a point cloud to best match a reference point cloud by reducing absolute vertical and horizontal errors [32]. ICP has been applied in geosciences e.g., for landslide and tectonic mapping [31,33]. ICP processing was performed using the point cloud alignment tool (pc_align) within the NASA Ames Stereo Pipeline (ASP) [34]. This was done separately for each snow-on DEM by using only the snow-free areas (assumed to be stable areas). The snow-free areas were manually delineated from the ortho-rectified camera images. The pc_align utility was configured with the “highest-accuracy” option and the alignment method was set to “similarity-least-squares” [34].

Experiments 2 and 3 differ by the source imagery of the snow-off DEM. For Exp. 2, a snow-off DEM was produced from photographs acquired on 28 November 2016 using the cameras when the scene was snow-free. For Exp. 3, a snow-off DEM was produced from an unmanned aerial vehicle (UAV) photogrammetric survey. In both cases, the DEMs were also computed in Agisoft Photoscan and accurately georeferenced using GCPs. For the snow-off DEM that was made from the terrestrial cameras images, the same six GCPs as above (Figure 3) were used. For the UAV snow-off DEM, another 6 GCPs were collected using a GPS device.

The computation of the differences between the co-registered snow-on DEMs and snow-off DEM was done using the geo-diff tool in ASP (step 4). This gave us a raster map of the elevation differences by pixel (dDEM), which can be compared to field measurements of the snow depth. Finally, the snow volume in the sinkhole was computed by spatial integration of the dDEM within the snow cover area polygon. Table 1 summarizes the different experiments that were conducted to compute the snow-on surface elevation for six validation dates.

### 3.3. Ground Truth Measurements

**Figure 4:**
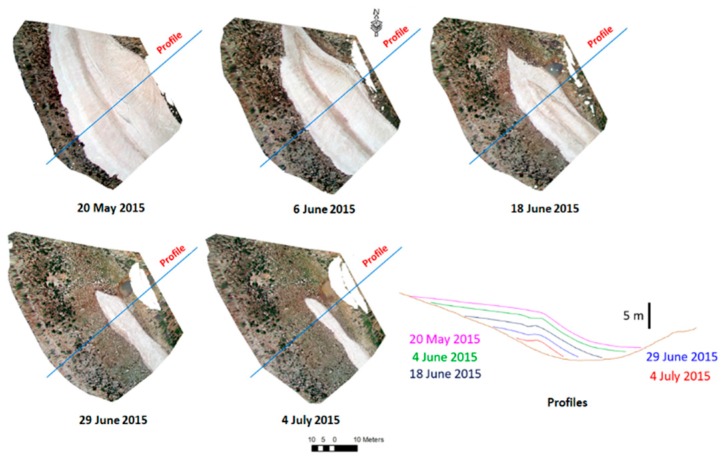
Profiles of the snow surface extracted from the DEM produced from the total station data total station.

The elevation of the snow surface in the study area was surveyed on 20 May 2015, 4 June 2015, 18 June 2015, 29 June 2018, and 4 July 2015 (Figure 4) using a total station (Leica TS06). The total stations have been, until now, one of the main instruments used for obtaining high accuracy surveying measurements, including topography, hydrography, construction, geography, hydrology, and many other fields [35,36]. As an example, the total stations have also validated the accuracy of the United States geological digital elevation models (DEMs) [35].

**Figure 5:**
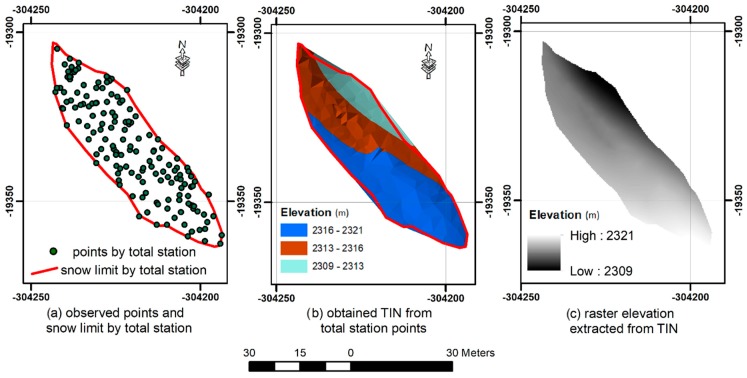
Steps to obtain a raster DEM from total station points.

Although, measurements with a total station do not provide a dense cloud of the surveyed area, it is possible to obtain centimeter to millimeter accuracy of any observed point position. Hence, the points obtained by the total station were used as reference altitudes to validate the DEM that were obtained from the terrestrial photogrammetry. On each date, the random points were measured on the snow cover. The criteria for choosing the points locations was a space of around 5 m for a flat area; the points were densified to approximately 1 m, when a change of slope was noticed. In other words, the locations of the points were randomly selected, but the spacing between he points varied, depending on the topography (closer points where the snow surface was more variable). The number of measured points for each date is shown in Table 2. For the comparison of the total station DEM with the camera DEM, the total station points were converted to a raster, with the same spatial resolution of 10 cm. The points were first converted to triangular irregular network (TIN) and then to a regular elevation grid in ArcMap software (Figure 5).

During the same topographic surveys, the limit of the snow cover was marked using a specific code when acquiring the point coordinates on the edge of the snow patch. These points were assembled as a single polygon to delineate the snow limit.

## 4. Results

**Figure 6:**
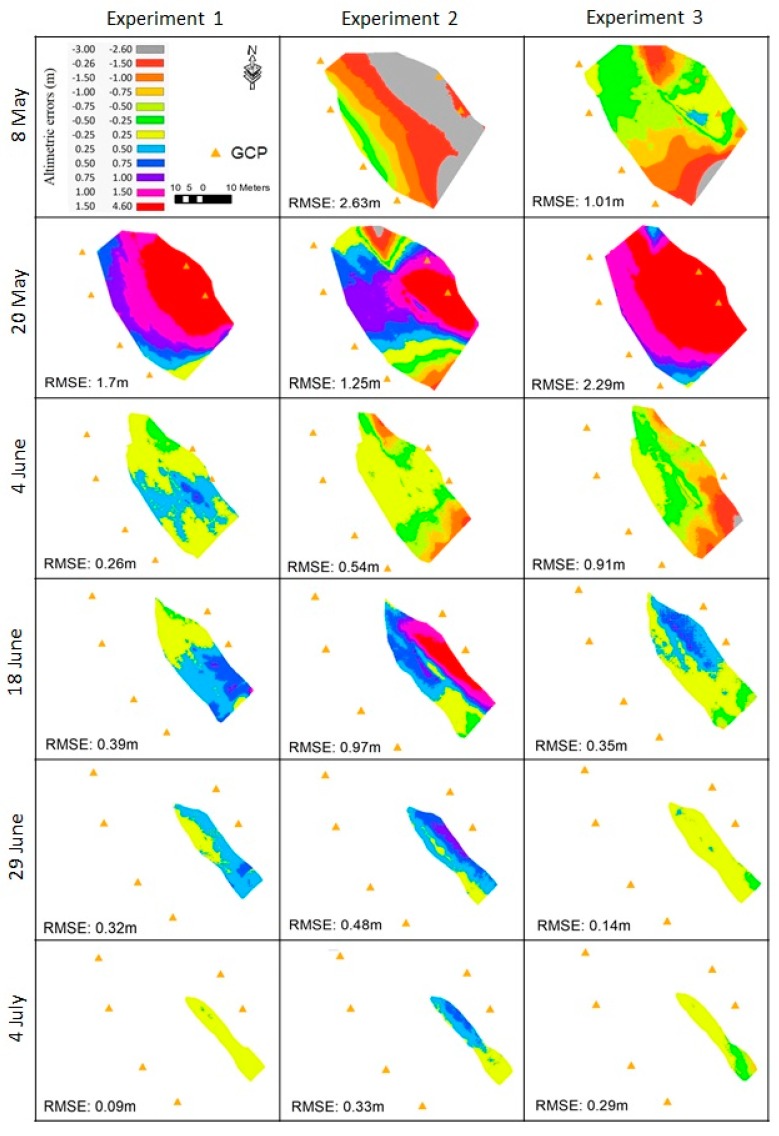
Maps of the elevation difference between total station data and camera DEM for each date and experiment.

**Figure 7:**
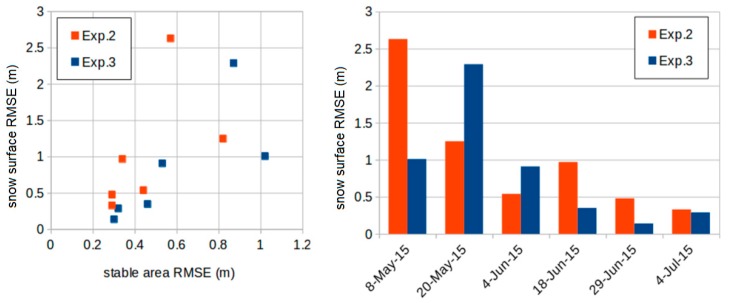
(**Left**) scatterplot of the RMSE (root mean square error) of the elevation on the stable areas (snow-free) and the RMSE on the snow surface, (**Right**) evolution of the RMSE on the snow surface with time.

**Figure 8:**
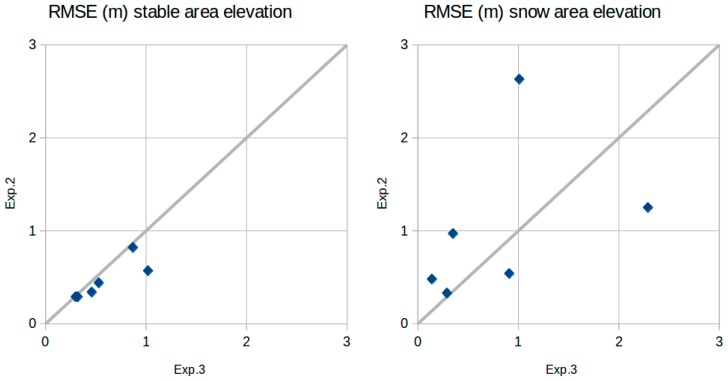
Comparison of the RMSE on the surface elevation from Exp.2 and Exp.3 over the snow surface and the stable area.

We present here the results of the validation for each experiment (Table 1). The snow-on DEMs were compared to the total station measurements. Figure 6 shows the spatial variability in the elevation residuals and Table 3 provides the statistical description of the residuals. The results indicate that the snow depth was retrieved with accuracy (as expressed by the standard deviation of the residuals) between 0.08 and 1.4 m, depending on the date and the method (median value 0.31 m). Except for the first two dates, the absolute bias is generally below 1 m for all experiments (median of the absolute bias values: 0.35 m). The bias is below 50 cm in 10 out of 17 cases. The root mean squared error (RMSE) values on the snow surface elevation range between 2.63 m for Exp. 2 DEM, that was conducted on 5 May 2015, to 0.09 m for the Exp. 1, which was conducted on 4 July 2015. Similarly, in relation to bias, the large range of RMSE is mainly explained by the date of the experiment (Figure 7). In particular, low RMSE values were obtained at the end of the snow season (04-Jun-2015, 29-Jun-2015, and 4-July-2015). For Exp. 2 and Exp. 3, a reduction of the RMSE through the snow season was observed on both, the stable area elevation and the snow surface elevation (Figure 8). Figure 6 shows that the spatial distribution of the error is consistent between Exp. 2 and Exp. 3, which suggests that the elevation errors are primarily due to the snow-on DEMs, since only the snow-off DEMs differ between both experiments.

The results also indicate that the accuracy on stable area elevation is slightly better with Exp. 3 than Exp. 2 (Figure 8). However, this does not mean there was better accuracy for the snow surface elevation, since the RMSE on the snow surface elevation is not systematically higher with Exp. 2.

## 5. Discussion

**Figure A1:**
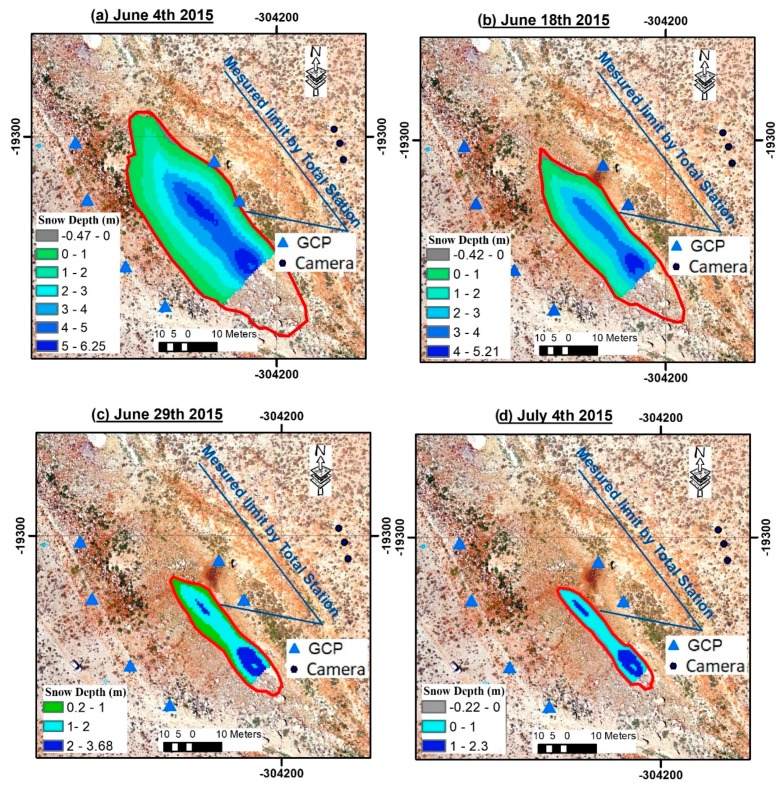
Snow depth maps of Exp.1.

**Figure A2:**
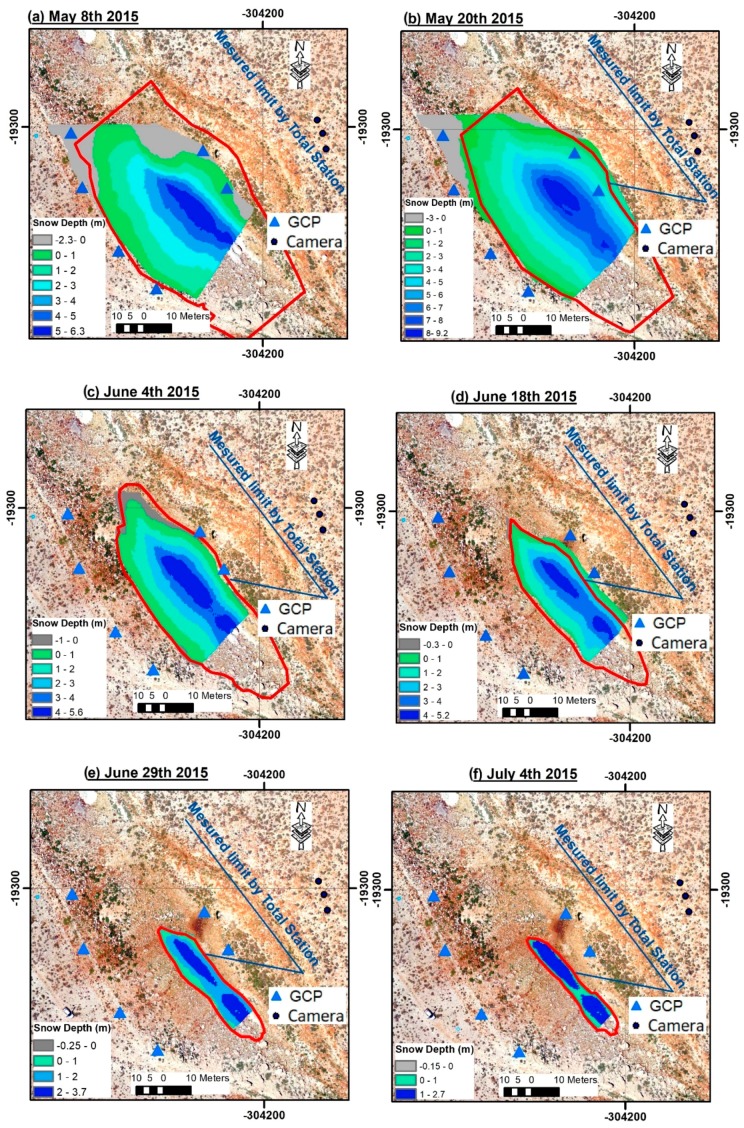
Snow depth maps of Exp.2.

**Figure A3:**
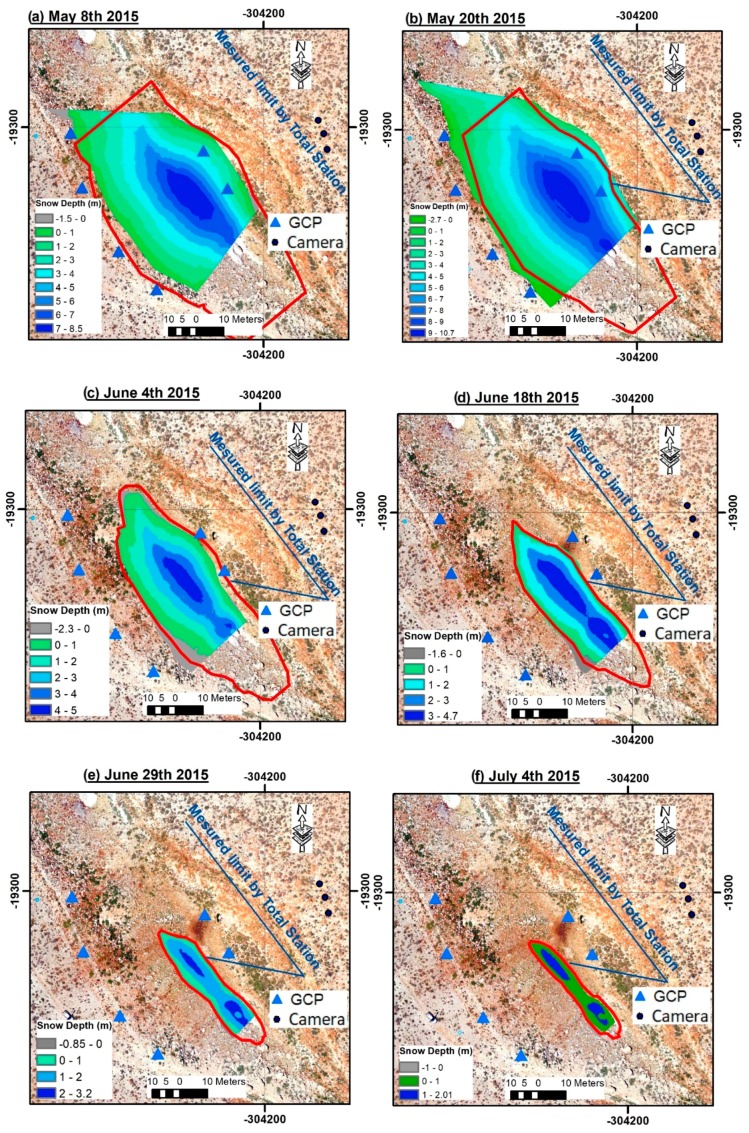
Snow depth maps of Exp.3.

The fact that the largest biases were found at the beginning of the season is clearly linked to the co-registration method (step 3, Section 3.2). Indeed, for Exp. 2 and Exp. 3, the RMSE on the stable area elevation also decreases when the RMSE on the snow surface elevation decreases (Figure 7). This can be understood by the fact that the snow-free areas, used in step (3), are located near the edges of the camera photographs where the deformation is expected to be maximal (Figure 6). Hence, at the beginning of the snow season, the stable terrain area is too limited to allow a proper alignment of the snow-on and snow-off DEMs. Our workflow provides acceptable results after 20 May 2015, when the snow covered area is not too large to allow a proper alignment between the snow-on and snow-off DEMs. However, despite a smaller snow covered area, there was still considerable snow after that date, since the maximum snow height on 4 Jun-2015, was 5.6 m (Figure A1, Figure A2, Figure A3).

The fact that Exp. 3 provided a better representation of the stable terrain was expected, since the drone images are less oblique than the cameras images. Interestingly, the results suggest that this does not necessarily improve the accuracy on the snow surface elevation, meaning that a drone survey to create a snow-off DEM might not be necessary for another installation. This could be due to the fact that the snow-off camera DEM was generated with photographs having the same acquisition geometry than as the snow-on DEMs, looking toward the main slope of the sinkhole. In this region, the acquisition geometry yields better DEM resolution, due to the steep slope facing the cameras. Since this is the area where most of the snow accumulates, the error on the snow elevation values is not affected by the source of the snow-off DEM.

Several factors could further affect the accuracy of the estimated snow depth, including the lens distortion and camera orientation, which cannot be fully corrected by the structure-from-motion algorithm, given the low number of photographs. The theory of structure-from-motion suggest that a higher accuracy could be achieved using more cameras, but this would increase the cost and the work-load to maintain the system. It is also likely that, more expensive cameras could provide better results especially where digital cameras, with higher dynamic range, may better resolve the texture variations over the snow surface. This would help the image correlation step within the structure-from-motion processing. However, it should be noted that the present accuracy between 20 and 60 cm (standard deviation of the residuals) is already acceptable, given that the observed snow depth ranges between 0 and 8 m in May, and between 0 and 5 m in early June. The error only becomes relatively significant in late June/July when the snow depth ranges between 0 and 2 m (Figure A1, Figure A2 and Figure A3 in Appendix A).

## 6. Conclusions

We have studied the accuracy of a new low-cost system to monitor the snow depth in a sinkhole, in the snow-dominated area of Mount-Lebanon. The system is based on three standard compact digital camera photographs, which typically cost USD $100 each. The cost of the system can be further reduced by using a single-board microcontroller instead of the CR200 data-logger, in order to trigger image acquisition.

The results indicate that a simple workflow, using Agisoft Photoscan and Ames Stereo Pipeline software, can estimate the snow surface elevation, with an acceptable accuracy between 20 and 60 cm (standard deviation of the residuals), and a low bias (below 50 cm) when there is sufficient stable terrain around the snow patch. We observed that the co-registration worked well with a ratio of stable area to snow area of roughly one third, but this is probably highly-dependent on the distribution of the stable terrain across the scene. The Agisoft Photoscan software is commercial, and was used in this study because it is efficient and user-friendly, however, open-source alternatives exist to perform the computation of the 3D model with similar accuracy (e.g., MicMac [37]).

The system is autonomous and requires only minimal maintenance. It can be fully automated to generate a time series of snow surface DEMs, as the processing is not based on field data, apart from the coordinates of the cameras that need to be surveyed once. In addition, it was possible to setup a near real-time system by transmitting the cameras images using mobile telecommunications networks. This could be useful in supporting the monitoring of the water resource, since the snow volume in the sinkhole can be considered a proxy of the state of the snow resources in the Mzaar area. Our future work will focus on the generation and analysis of the daily time series of snow volume in the sinkhole over three complete snow seasons from these time-lapse cameras.

## Figures and Tables

**Table 1:** Characteristics of the three experiments to compute the snow-on surface elevation (GCP: ground control point, ICP: iterative closest point).

	Snow-off DEM Source Imagery	Snow-on DEM Source Imagery	Method to Register Snow-on DEM
Exp. 1	cameras	cameras	GCP
Exp. 2	cameras	cameras	ICP
Exp. 3	drone	cameras	ICP

**Table 2:** Number of points measured by the total station for each surveyed day.

Date	Number of Points
8 May 2019	210
20 May 2019	250
4 June 2019	219
18 June 2019	140
29 June 2019	168
4 July 2019	92

**Table 3:** Statistics of the residuals between the snow-on DEMs and the field measurements. The RMSE over the stable area is provided as an indicator of the performance of the ICP adjustment. The results are not available for Exp. 1 because the GCPs were covered by the snow are thus not visible in the photographs.

Date	Exp.	Min (m)	Max (m)	Mean (m)	Std (m)	RMSE (m)	Stable Area RMSE (m)
8-May-2015	1	N/A	N/A	N/A	N/A	N/A	N/A
	2	−6.78	0.09	−2.23	1.40	2.63	0.57
	3	−3.32	0.53	−0.79	0.63	1.01	1.02
20-May-2015	1	−0.21	3.39	1.50	0.81	1.70	
	2	−3.10	3.35	0.66	1.06	1.25	0.82
	3	−0.06	4.45	2.05	1.03	2.29	0.87
4-Jun-2015	1	−0.57	0.81	0.17	0.20	0.26	
	2	−1.86	0.32	−0.35	0.41	0.54	0.44
	3	−3.12	0.11	−0.73	0.54	0.91	0.53
18-June-2015	1	−0.46	1.44	0.28	0.27	0.39	
	2	−0.54	2.2	0.74	0.63	0.97	0.34
	3	−0.94	1.00	0.17	0.31	0.35	0.46
29-June-2015	1	−0.03	0.77	0.30	0.12	0.32	
	2	−0.10	0.91	0.43	0.21	0.48	0.29
	3	−0.48	0.33	0.02	0.14	0.14	0.30
4-July-2015	1	−0.18	0.33	0.03	0.08	0.09	
	2	−0.22	0.68	0.27	0.19	0.33	0.29
	3	−0.96	0.27	−0.15	0.25	0.29	0.32
